# In Vitro Antibacterial Activity of Galls of *Quercus infectoria* Olivier against Oral Pathogens 

**DOI:** 10.1155/2012/632796

**Published:** 2011-11-17

**Authors:** Dayang Fredalina Basri, Liy Si Tan, Zaleha Shafiei, Noraziah Mohamad Zin

**Affiliations:** ^1^Department of Biomedical Science, Faculty of Health Sciences, Universiti Kebangsaan Malaysia, Jalan Raja Muda Abdul Aziz, 50300 Kuala Lumpur, Malaysia; ^2^Department of Clinical Oral Biology, Faculty of Dentistry, Universiti Kebangsaan Malaysia, Jalan Raja Muda Abdul Aziz, 50300 Kuala Lumpur, Malaysia

## Abstract

The galls of *Quercus infectoria* are commonly used in Malay traditional medicine to treat wound infections after childbirth. In India, they are employed traditionally as dental applications such as that in treatment of toothache and gingivitis. The aim of the present study was to evaluate the antibacterial activity of galls of *Quercus infectoria* Olivier against oral bacteria which are known to cause dental caries and periodontitis. Methanol and acetone extracts were screened against two Gram-positive bacteria (*Streptococcus mutans* ATCC 25175 and *Streptococcus salivarius* ATCC 13419) and two Gram-negative bacteria (*Porphyromonas gingivalis* ATCC 33277 and *Fusobacterium nucleatum* ATCC 25586). The screening test of antibacterial activity was performed using agar-well diffusion method. Subsequently, minimum inhibitory concentration (MIC) was determined by using twofold serial microdilution method at a concentration ranging between 0.01 mg/mL and 5 mg/mL. Minimum bactericidal concentration (MBC) was obtained by subculturing microtiter wells which showed no changes in colour of the indicator after incubation. Both extracts showed inhibition zones which did not differ significantly (*P* < 0.05) against each tested bacteria. Among all tested bacteria, *S. salivarius* was the most susceptible. The MIC ranges for methanol and acetone extracts were the same, between 0.16 and 0.63 mg/mL. The MBC value, for methanol and acetone extracts, was in the ranges 0.31–1.25 mg/mL and 0.31–2.50 mg/mL, respectively. Both extracts of *Q. infectoria* galls exhibited similar antibacterial activity against oral pathogens. Thus, the galls may be considered as effective phytotherapeutic agents for the prevention of oral pathogens.

## 1. Introduction

Dental caries and periodontal disease are prevalent worldwide. In most industrialized countries, dental caries affects 60–90% of school-aged children as well as the vast majority of adults [1]. Poor oral health affects the general population, and it is often related to chronic disease like diabetes [1]. Bacteria existing in the dental plaque or biofilm play an important role in the development of both dental caries and periodontal disease [2]. 

The approach to prevent such oral diseases is targeted at the control of dental plaque formation [3]. However, antimicrobial agents that have been widely used today can result in changes on oral microbiota and even produce teeth staining by chlorhexidine mouth rinse [4]. In addition, oral bacteria have been reported to show increased resistance towards common antibiotics such as penicillin, cephalosporin, erythromycin, tetracycline, and metronidazole which have been used therapeutically for the treatment of oral infection [5]. The increase in resistance and adverse effects have lead researchers to explore novel anti-infective herbal compounds which could be used for effective treatment of oral diseases. 

Medicinal plants have been used in traditional treatment in various parts of the world, especially in rural areas [6]. Approximately 80% of the population in developing countries still use traditional medicines for their health care [7]. The natural products derived from medicinal plants are known to produce biologically active compounds. 

The galls of *Quercus infectoria* Olivier (Fagaceae) are locally known as “biji manjakani” in Malaysia, and it is one of the most popular traditional medicine in Asia. *Quercus infectoria* is a small tree or shrub about 2 m high and mainly found in Turkey, Syria, Persia, Cyprus, and Greece [8]. The galls arise on the twigs of this tree resulting from the deposition of eggs by gall wasp *Cynips gallae tinctoriae *[9]. The main constituents of the galls are gallotannic acid (50–70%), gallic acid (2–4%), ellagic acid, starch, and sugar [10]. In Malaysia, the galls have been used in Malay traditional medicine to restore postpartum uterine elasticity and also stimulate the contraction of vaginal muscles [11]. In Indian traditional medicine, it is a constituent of toothpowder or toothpaste for treatment of gum and oral cavity diseases [12]. The galls have been used to treat diarrhea, dysentery, internal hemorrhages, gonorrhea, impetigo, tonsillitis, and menorrhagia [13]. Pharmacologically, the galls have been reported to possess activities such as antidiabetic, antibacterial, antiviral, antifungal, larvicidal, anti-inflammatory, antiamoebic, and wound healing [14–21]. 

To the best of our knowledge, no past researches have been conducted on methanol and acetone extracts of the *Quercus infectoria* galls against the Gram-negative bacteria. Keeping in view the antibacterial properties of the galls of *Quercus infectoria*, the present study was carried out to investigate the action of the gall extracts against oral pathogens. 

## 2. Materials and Methods

### 2.1. Plant Materials

The galls of *Q. infectoria* were purchased from the local market and were identified at the *Forest Research Institute Malaysia* (FRIM) with voucher number D3-ZR/0023. Pestle and mortar was used to crush the washed and dried galls before being powdered in an electric grinder. 

### 2.2. Preparation of Crude Extract

The dried gall powder was extracted by using methanol (Scharlau Chemie S.A., Spain). It was prepared by cold extraction technique [15]. In the ratio of 1 : 5, 100 g of the dried gall powder was immersed in 500 mL methanol for 24 hours at room temperature. The mixture was then filtered by using the Whatman no. 1 filter paper, and the filtrate was stored. The process was repeated by using the remaining residue with 300 mL methanol. Both filtrates were then mixed and concentrated under reduced pressure by using a rotary evaporator (Eyela N-1000, Japan). The resulting pellet was finally pounded to dryness under hot air dryer to produce a powdery crude methanol extract; the acetone extract was obtained from the remaining sample. 

### 2.3. Preparation of Extract Solution

The extracts were dissolved in sterile distilled water to a final concentration of 100 mg/mL for agar-well diffusion method and 20 mg/mL for broth microdilution method. All the extracts were sterilized by passing through a 0.45 *μ*m membrane filter (Fisher Scientific Co., USA).

### 2.4. Microorganisms

The bacterial species used in this study were two facultative anaerobic Gram-positive bacteria, which were *Streptococcus mutans* ATCC 25175 and *Streptococcus salivarius* ATCC 13419 as well as two obligate anaerobic Gram-negative bacteria which were *Porphyromonas gingivalis* ATCC 33277 and *Fusobacterium nucleatum *ATCC 25586. The facultative anaerobes were grown on brain heart infusion (BHI) (Lab. M, UK) agar slants which were supplemented with yeast extract, hemin, and vitamin K (S-BHI) and used for obligate anaerobes. All the agar slants were incubated in 5% CO_2_ incubator (Shel Lab 2424-2, USA) for 24 hours (facultative anaerobes) or 48 hours (obligate anaerobes in anaerobic jar) at 37°C. The agar slants with bacterial growth were stored in refrigerator at 4°C for usage within 3 months. 

The inoculum size of each bacterial species (10^8^ bacteria/mL for agar-well diffusion method) was standardized by using spectrophotometer (Metertech SP-830, Taiwan). The turbidity of the bacterial suspension was adjusted to absorbance (A) reading within the range of 0.08 to 1.00 at 625 nm [22]. 

### 2.5. Screening of Antibacterial Activity

The agar-well diffusion method was used to evaluate the antibacterial activity of all the extracts from the *Q. infectoria* gall. It was performed by modification of a previous method [23]. This assay for each bacterial species used the same method as that of the growth media. Each agar plate was uniformly seeded with bacteria by means of sterile swab dipped in the standardized suspension and streaked on the agar plate surface. Wells of 5 mm in diameter were punched into the inoculated agar media with sterile Pasteur pipette. Approximately, 50 *μ*L of the extract solution were dropped into each well which filled them, respectively, to fullness. For positive control, chloramphenicol disc (30 *μ*g/mL) was used for Gram-positive bacteria, whereas tetracycline disc (30 *μ*g/mL) was used for Gram-negative bacteria. Distilled water served as negative control. The plates were incubated in anaerobic condition at 37°C overnight (facultative anaerobes) or for 48 hours (obligate anaerobes). The antibacterial activity was interpreted from the size of inhibition zones diameter which were measured in mm from observation of clear zones surrounding the wells. Each extract was assayed in triplicate in order to calculate the mean value. 

### 2.6. Determination of MIC and MBC Values

The minimum inhibitory concentration (MIC) value of each extract was determined for all the bacterial strains by using the twofold serial microdilution method which was done in 96-well microtiter plate. This assay was performed by modification of a previous method [24]. Initially, the tested extract (50 *μ*L, 20 mg/mL) was added to the sterile broth media (50 *μ*L) which were similar to the media used in the screening test. Subsequently, 50 *μ*L diluted bacterial suspension with final inoculum of 10^5^ bacteria/mL was added to the microtiter plate. The microdilution was performed at a final concentration (0.01 mg/mL–5 mg/mL). Each extract was assayed in triplicate. The extracts in broth were used as negative control to ensure medium sterility while the bacterial suspensions served as positive control to control the adequacy of the broth for bacterial growth. Following incubation under anaerobic conditions, the plate was added with 20 *μ*L 3-[4,5-dimethylthiazol-2-yl]-2,5-diphenyltetrazolium bromide (MTT, 1 mg/mL) for Gram-positive bacteria and 40 *μ*L 2,3,5-triphenyltetrazolium chloride (TTC, 2 mg/mL) for Gram-negative bacteria. The plate was then incubated again for another 2 hours in the dark. The MIC value was taken as the lowest concentration of extract that showed no color changes of indicator after addition. 

The minimum bactericidal concentration (MBC) value was determined by subculture of the wells which showed no color changes on the sterile agar plate. The least concentration which showed no visible growth on agar plate was considered as MBC value. 

### 2.7. Statistical Analysis

SPSS version 19 was used for statistical comparison of the mean value for inhibition zone obtained from extract and positive control. One-way ANOVA followed by post hoc Tukey HSD was used for Gram-positive bacteria while the Kruskal-Wallis followed by the Mann-Whitney Utests was used for Gram negative bacteria.

## 3. Results 

### 3.1. Screening of Antibacterial Activity

The antibacterial activity of methanol and acetone extracts from galls of *Q. infectoria* against each oral bacteria species were tabulated (Table 1). Both extracts exhibited inhibitory effects which were not significantly different (*P* < 0.05) against each bacterial species tested. In other words, the size of inhibitory zones showed by methanol and acetone extracts did not differ significantly against each of the bacteria tested: 22.67 ± 0.33 and 21.33 ± 0.33, respectively, against *S. mutans*, 25.33 ± 0.33 and 24.33 ± 0.33, respectively, against *S. salivarius*, 18.33 ± 0.33 for both extracts against *P. gingivalis*, 18.67 ± 0.33 and 19.33 ± 0.33, respectively, against *F. nucleatum*. Among all the bacteria, *S. salivarius* was found to be the most susceptible towards both extracts and even displayed significantly larger inhibition zone compared to positive control. Inhibition zones shown by both extracts against other bacteria were significantly lesser compared to the positive control. The smallest inhibition zone was shown by both extracts against* P. gingivalis*. Overall, all the extracts from the gall exhibited a moderately stronger inhibitory effect against Gram-positive bacteria compared to the Gram-negative bacteria. 

### 3.2. Determination of MIC and MBC Values

The MIC values of the methanol and acetone extracts from galls of *Q. infectoria* against all oral bacteria strains were observed (Table 2). Interestingly, both extracts were found to exhibit similar MIC values against each bacteria species, ranging from 0.16 to 0.63 mg/mL. The MIC values of both extracts correlated to the screening test result. *S. salivarius* appeared to be the most susceptible bacteria with the lowest MIC value among all the bacteria species tested. *S. mutans* and *P. gingivalis* displayed the same MIC values.

The MBC values of the methanol and acetone extracts from galls of *Q. infectoria* against all oral bacteria species were observed (Table 3). Methanol extract displayed higher MBC value (0.31 mg/mL) compared to MIC value (0.16 mg/mL) against *S. salivarius*. This was also observed on *P. gingivalis* where its MBC value (1.25 mg/mL) was higher than MIC value (0.63 mg/mL). It was observed that methanol extract showed MBC value similar to MIC value against *S. mutans*, which was 0.63 mg/mL. For *F. nucleatum*, MBC and MIC values for both extracts were the same (0.31 mg/mL). Acetone extract displayed much higher MBC value (2.5 mg/mL) than MIC value against both *S. mutans* and *P. gingivalis*. For *S. salivarius*, the MBC value (0.63 mg/mL) was also found to be higher than the MIC value. 

## 4. Discussion

Over the years, traditional healers have used water primarily as solvent. Plant extracts dissolved in organic solvents such as methanol are reported to exhibit more antibacterial activity compared to the aqueous extract [25]. Moreover, acetone is a better extractor, followed by methanol, ethanol, and water [26]. Acetone is a useful extractant because it dissolves wide range of active compounds from plants including both hydrophilic and hydrophobic components as well as low toxicity to test organisms [27]. Beside that, use of organic solvent as extractant does not give negative effect on their bioactivity against bacteria tested [28].

In the present study, methanol and acetone extracts were found to possess the ability to inhibit growth of all oral. This indicates that the extract of galls of *Q. infectoria* contains broad-spectrum antibacterial compounds which make it a potentially good source of antimicrobial substance. Furthermore, both extracts exhibited greater inhibitory effect on Gram-positive bacteria compared to Gram-negative bacteria. This finding was in accordance with previous work which had reported that the growth of Gram-positive bacteria was easily inhibited by natural plant extracts compared to Gram-negative bacteria [29]. The reason for this difference is attributed to the difference in bacterial cell wall composition [30].

Inhibitory activities of both extracts did not show significant difference towards each bacterial species tested. This may be indicative of the presence of hydrophilic antibacterial compounds in the extract since both solvents could extract out polar substances from plant materials. Specifically, both extracts appeared to react most sensitively against *S. salivarius*. Furthermore, the most interesting finding was that inhibition zone (value) showed by the positive control was significantly smaller compared to those displayed by both extracts against *S. salivarius*. This suggests that the bacteria species was more susceptible to the extracts compared to the commercial antibiotic. Results of antibacterial activity shown on *S. mutans* and *S. salivarius* are similar to previous finding which tested methanol extract of galls on both bacteria [24]. The strong antibacterial activities of methanol extract against *S. mutans* have been reported previously [31, 32]. On the other hand, the present study was the first study of its kind which showed the inhibitory effect of galls of *Q. infectoria* extracts towards both *P. gingivalis* and* F. nucleatum* which constitute obligate anaerobic bacteria group. 

For MIC value determination, methanol and acetone extracts showed similar MIC values against each bacteria species tested. This finding indicated that both extracts displayed same potency for all bacteria tested. The MIC values of both extracts were correlated to screening test result where both extracts were more potent against *S. salivarius* with lowest MIC value. All the past studies looked into the screening test, but, to the best of our knowledge, none of the studies compared the MIC values with other plant extracts. Compared to methanol extract from *Ficus carica* (fig) leaves, the methanol extract from the gall of *Q. infectoria* was found to be more active against *S. mutans *and *F. nucleatum* because of the lower MIC values [33]. Both plant extracts showed the same MIC values against *P. gingivalis*. The different antimicrobial activity of both plant extracts may be due to the presence of different active compounds. The leaves of *Ficus carica* contain flavonoid which acts as an antimicrobial substance [33]. In fact, both tannin and flavonoid have been reported to exhibit antibacterial activity on different strains of *S. mutans *[34]. 

In order to characterize types of antibacterial activity displayed by a plant extract, MBC value determination is necessary so that comparison of both MIC and MBC values can be made. Knowing about bacteriostatic or bactericidal activity exhibited by a plant extract against bacterial species is important in screening of a new antimicrobial agent [35]. In this study, both methanol and acetone extracts showed bacteriostatic activity on *S. salivarius* and *P. gingivalis* while bactericidal activity was shown against *F. nucleatum*. For *S. mutans*, bactericidal and bacteriostatic activities were shown by methanol and acetone extracts, respectively. The difference of antibacterial activity exhibited on the bacteria species tested was due to the different susceptibility of each bacteria species which reacted with gall extract. However, this comparison only gives a description of the antibacterial activity. Detailed study which is time-kill study needs to be carried out to determine accurately the bactericidal activity shown by the extract [36]. 

There is no previous study that showed the types of antibacterial activity of gall extract against oral bacteria. However, this activity was exhibited by hydrolysable tannin present in other plant extracts. For instance, bactericidal activity of gallotannin from *Melaphis chinensis *Bell (Chinese gall) against *S. mutans* had been reported [37]. Besides, methyl gallate and gallic acid, which were the main components of gallotannin from Galla Rhois wu be zi (outgrowth of *Rhus chinensis *Mill), were found to prevent growth (bacteriostatic) of cariogenic bacteria as well as periodontal bacteria [38] tested in this study. 

High amounts of hydrolysable tannin present in the galls of *Q. infectoria* implied that tannin may be the active compound responsible for the antibacterial activity in this study. Tannins in the galls were reported to possess antibacterial property against common pathogens such as *Enterococcus faecalis*, *Streptococcus pyogenes,* and *Bacillus cereus *[39]. The antimicrobial activity seemed to depend on total tannin content in the plant extracts [40]. The antibacterial activity of extract from *Areca catechu *Linn. against *S. mutans*, *S. salivarius,* and *F. nucleatum* is due to the presence of hydrolysable tannin in that extract [41]. A number of mechanisms have been proposed to explain the antibacterial activity shown by tannin such as complex formation between tannin and microbial enzymes (such as cellulase) as well as membrane of microorganism due to the astringent properties of tannin, iron deprivation through precipitation and effect on bacterial metabolism through inhibition of oxidative phosphorylation [42]. 

## 5. Conclusion 

This study has proved the high potential of the extracts from galls of *Q. infectoria* to resist the growth of oral bacteria. It also provides an insight into the usage of these galls in traditional treatment of oral disease associated with bacterial infection. Besides, it can be used effectively as a supplementary agent in clinical treatment of periodontal disease. However, further investigations are needed to study in detail the effect of the gall extracts on activities involved in dental caries formation and understand the pathogenesis of periodontitis. 

## Figures and Tables

**Table 1:** Inhibition zones diameter of extracts from galls of *Q. infectoria *against oral bacteria.

Bacterial	Inhibition zone diameter (mm ± SEM)			
species	Methanol extract	Acetone extract	Positive control	Negative control
*S. mutans *	22.67 ± 0.33*	21.33 ± 0.33*	25.67 ± 0.33	−
ATCC 25175				
*S. salivarius *	25.33 ± 0.33*	24.33 ± 0.33*	22.33 ± 0.33	−
ATCC 13419				
*P. gingivalis *	18.33 ± 0.33*	18.33 ± 0.33*	20.00 ± 0.00	−
ATCC 33277				
*F. nucleatum *	18.67 ± 0.33*	19.33 ± 0.33*	25.00 ± 0.00	−
ATCC 25586				

*Significant difference as compared with positive control (*P* < 0.05).

−: No inhibition zone.

**Table 2:** MIC values of methanol/acetone extract from galls of *Q. infectoria *against oral bacteria.

Concentration (mg/mL)	Bacterial species				Control	
	*S. mutans*	*S. salivarius*	*P. gingivalis*	*F. nucleatum*	Positive	Negative
	ATCC	ATCC	ATCC	ATCC		
	25175	13419	33277	25586		
5.00	−	−	−	−	+	−
2.50	−	−	−	−	+	−
1.25	−	−	−	−	+	−
0.63	−	−	−	−	+	−
0.31	+	−	+	−	+	−
0.16	+	−	+	+	+	−
0.08	+	+	+	+	+	−
0.04	+	+	+	+	+	−
0.02	+	+	+	+	+	−
0.01	+	+	+	+	+	−

+: Presence of indicator color changes.

−: Absence of indicator color changes.

**Table 3:** MBC values of methanol and acetone extracts from galls of *Q. infectoria *against oral bacteria.

Bacterial species	Extracts	Extract concentration (mg/mL)					
		5.00	2.50	1.25	0.63	0.31	0.16
*S. mutans* ATCC 25175	Methanol	−	−	−	−	ND	ND
	Acetone	−	−	+	+	ND	ND
*S. salivarius* ATCC 13419	Methanol	−	−	−	−	−	+
	Acetone	−	−	−	−	+	+
*P. gingivalis* ATCC 33277	Methanol	−	−	−	+	ND	ND
	Acetone	−	−	+	+	ND	ND
*F. nucleatum* ATCC 25586	Methanol	−	−	−	−	−	ND
	Acetone	−	−	−	−	−	ND

+: Presence of bacterial growth.

−: Absence of bacterial growth.

ND: not done because the microtiter well at the tested concentration showed the presence of bacterial growth as shown in Table 2.
